# Development and Actionability of the Dutch COVID-19 Dashboard: Descriptive Assessment and Expert Appraisal Study

**DOI:** 10.2196/31161

**Published:** 2021-10-12

**Authors:** Véronique L L C Bos, Tessa Jansen, Niek S Klazinga, Dionne S Kringos

**Affiliations:** 1 Department of Public and Occupational Health Amsterdam UMC Amsterdam Public Health Research Institute Amsterdam Netherlands; 2 Netherlands Institute for Health Services Research (Nivel) Utrecht Netherlands

**Keywords:** COVID-19, dashboard, performance intelligence, Netherlands, actionability, communication, government, pandemic, public health

## Abstract

**Background:**

Web-based public reporting by means of dashboards has become an essential tool for governments worldwide to monitor COVID-19 information and communicate it to the public. The actionability of such dashboards is determined by their fitness for purpose—meeting a specific information need—and fitness for use—placing the right information into the right hands at the right time and in a manner that can be understood.

**Objective:**

The aim of this study was to identify specific areas where the actionability of the Dutch government’s COVID-19 dashboard could be improved, with the ultimate goal of enhancing public understanding of the pandemic.

**Methods:**

The study was conducted from February 2020 to April 2021. A mixed methods approach was carried out, using (1) a descriptive checklist over time to monitor changes made to the dashboard, (2) an actionability scoring of the dashboard to pinpoint areas for improvement, and (3) a reflection meeting with the dashboard development team to contextualize findings and discuss areas for improvement.

**Results:**

The dashboard predominantly showed epidemiological information on COVID-19. It had been developed and adapted by adding more in-depth indicators, more geographic disaggregation options, and new indicator themes. It also changed in target audience from policy makers to the general public; thus, a homepage was added with the most important information, using news-like items to explain the provided indicators and conducting research to enhance public understanding of the dashboard. However, disaggregation options such as sex, socioeconomic status, and ethnicity and indicators on dual-track health system management and social and economic impact that have proven to give important insights in other countries are missing from the Dutch COVID-19 dashboard, limiting its actionability.

**Conclusions:**

The Dutch COVID-19 dashboard developed over time its fitness for purpose and use in terms of providing epidemiological information to the general public as a target audience. However, to strengthen the Dutch health system’s ability to cope with upcoming phases of the COVID-19 pandemic or future public health emergencies, we advise (1) establishing timely indicators relating to health system capacity, (2) including relevant data disaggregation options (eg, sex, socioeconomic status), and (3) enabling interoperability between social, health, and economic data sources.

## Introduction

In response to the COVID-19 pandemic, caused by the infection of the severe acute respiratory syndrome coronavirus 2 (SARS-CoV-2), that emerged in 2019, governments worldwide have been forced to take measures that impact the lives of individuals in order to protect the health of their citizens. Making the right COVID-19 policy decisions requires a balanced trade-off between protecting the population from infection and its consequences, ensuring that non-COVID-19 care needs are met (dual-track capacity monitoring), and minimizing socioeconomic impacts [1]. Web-based public reporting by means of dashboards has become an essential tool for monitoring COVID-19 information and communicating it to the public [2-4]. Moreover, dashboards are used to support individuals in informed decision making, for instance to educate citizens on whether they need to adapt their behaviors to minimize individual and population risk [5]. As such, dashboards are a powerful communication tool, and they are also frequently used by media as key information sources. If, however, information in dashboards is based on suboptimal reporting practices (eg, incomplete or unreliable reporting of data), it could produce undesirable effects, such as misleading perceptions, stress, or anxiety [6-9]. Users of dashboards must therefore be assured of complete, valid, reliable, and balanced information that can support them in making informed decisions as they deal with the pandemic. Information can be actionable only if it is fit for purpose and fit for use. Important determinations in the development of dashboards therefore include the selection and standardization of indicators at national and international levels, sources of data collection, analysis of data, and visualization techniques chosen to display the data [9,10]. The World Health Organization (WHO) advised 4 key types of information needed to effectively manage transitions and modulate restrictive measures over the course of the COVID-19 pandemic: (1) public health and epidemiological, (2) health system management, (3) behavioral insights, and (4) social and economic impact [11].

In mid-2020, an international network of performance intelligence researchers [12], to which the authors of this paper belong, carried out a global study to assess the actionability of 158 COVID-19 dashboards at international, national, and regional levels in 53 countries worldwide [13]. Actionability refers to a dashboard’s potential to inform decision making by way of providing information that is both *fit for purpose*—meeting a specific information need—and *fit for use*—placing the right information into the right hands at the right time and in a manner that can be understood [14]. The study identified 7 features that were common to highly actionable dashboards: (1) knowing the audience and their information needs; (2) managing the type, volume, and flow of displayed information; (3) reporting data sources and methods clearly; (4) linking time trends to policy decisions; (5) providing “data close to home”; (6) disaggregating the information into relevant subgroups; (7) using storytelling and visual cues.

The international dashboard study also found that there was overlap in indicators between the international dashboards, being that most dashboards portrayed case numbers, hospital admissions, and deaths due to COVID-19. Yet, little is currently known about how dashboards have improved their content and actionability over the course of the pandemic and what guided the decisions that were made in the development of the dashboards. This will very likely be context-dependent, thus a closer look at a specific country context can provide insight into the “why” of COVID-19 dashboard development. One study has looked at the development of Canadian COVID-19 dashboards and concluded that data availability and dashboard technology witnessed the most improvements. However, no improvements were found in communicative elements [15]. The development of the Dutch government’s COVID-19 dashboard, led by the Ministry of Health, Welfare and Sport, is continuously evolving in which publicly available feedback from expert panels [16] and from surveys among the public [17,18] are used to further develop the dashboard’s content and its communication strategy. This makes it an interesting case study for research. With this study, we set out to evaluate how the Dutch COVID-19 dashboard developed from its launch on June 5, 2020 through January 31, 2021, to assess its actionability, and to understand what factors guided decisions during the dashboard’s development.

Those objectives translated into the following research questions: (1) How did the Dutch COVID-19 dashboard develop over the course of the pandemic from its launch on June 5, 2020 to January 31, 2021? (2) How actionable is the Dutch COVID-19 dashboard, based on its December 2020 version, reflecting on features of highly actionable COVID-19 dashboards? (3) What decisions were made in the development of the COVID-19 dashboard by its developers and why?

This study aimed to improve the Dutch COVID-19 dashboard’s actionability in order to enhance public understanding of the pandemic.

## Methods

### Scope and Study Setting

This study focused on the development of the Dutch government’s COVID-19 dashboard [19] from June 5, 2020 (launch) to January 31, 2021. The first confirmed COVID-19 patient in the Netherlands was reported on February 27, 2020 to the House of Representatives by the Minister for Medical Care [20]. Hygiene and safety recommendations announced on March 9, 2020 at a press conference by the Dutch prime minister, accompanied by the director of the National Institute for Public Health and the Environment (RIVM), were rapidly followed by a number of policy measures to enforce physical distancing, impose travel restrictions, and establish case management and quarantine policies to contain the spread of the SARS-CoV-2 virus. In the weekly COVID-19 mortality numbers, a first peak was seen from mid-March to late May 2020, followed by relatively low numbers during the summer months of June to September, with numbers increasing again from October 2020 [21]. The COVID-19 measures taken by the government reflect these 2 high-burden periods, showing a pause or partial loosening for most measures in the June to September period [22,23]. In the management of the pandemic, the national government is advised by an Outbreak Management Team coordinated by the RIVM and works closely together with the 25 safety regions that, by law, have a responsibility for regional disaster management [24].

In May 2020, in addition to existing Dutch websites providing information on the pandemic [25-27], the Minister of Health, Welfare and Sport proposed a dedicated COVID-19 dashboard with the aim of keeping track of the pandemic and informing policy decisions to the House of Representatives [21]. On June 5, 2020, a test version of the official Dutch government dashboard for COVID-19 (entitled *Coronadashboard*) was launched for the general public [21].

### Descriptive Assessment of the Development of the COVID-19 Dashboard by the Netherlands Ministry of Health, Welfare and Sport Over Time

To examine the development of the Dutch COVID-19 dashboard, 3 points in time were chosen for our descriptive assessment. The first was conducted on July 16, 2020, approximately 1 month after the dashboard’s launch, and was part of our international study of COVID-19 dashboards [28]. The second was on December 23, 2020, amid new containment measures, and the third was on January 31, 2021, just after vaccinations had commenced. The dashboard website was archived at these time points (Multimedia Appendix 1). We used the descriptive checklist developed and validated in the international dashboard study (see Multimedia Appendix 2 for the checklist and its measures) [28]. The tool was based on communication theory (notably on Lasswell’s Model from 1948 [29]), the discipline of performance intelligence in health [30,31], and existing evidence on performance indicators, public reporting, and the use of dashboards [28]. In addition to general and context aspects, the tool focuses on whether the purpose of the dashboard and the type of users are noted, what indicators are presented, whether metadata and the sources are made available, what disaggregation options are presented, and the use of visualization techniques.

### Actionability Appraisal of the COVID-19 Dashboard

To appraise the actionability of the Dutch COVID-19 dashboard, we rated it in terms of the 7 common features of highly actionable COVID-19 dashboards (Table 1) [28].

**Table 1:** Seven common features of highly actionable dashboards.

Number	Feature^a^	Explanation
1	Knowing the audience and their information needs	Dashboards with a known audience and explicit aim had focus and continuity in their content, analysis, and delivery. Techniques such as guiding key questions or overall composite scores clearly communicated the decision they intended to support. Multilanguage functionality and exact timing of updating signaled an awareness and intent to encourage their regular use by the intended decision maker.
2	Managing the type, volume, and flow of displayed information	The selection of a concise number of indicators brought focus and importance to the information and the possibility to view indicators together at a glance. The use of indicators in moderation, although still spanning varied types of information, was especially effective. The ordering of information, from general to specific or in sections based on theme, made the flow of information intuitive.
3	Reporting data sources and methods clearly	A clear source of data and explanation of an indicator’s construction, including potential limitations, was found to be an important component of trust in the dashboard and clarity in its reporting. This information can be provided in short narratives that support users to understand what is in fact being presented.
4	Linking time trends to policy decisions	Reporting data over time together with the introduction of key infection control measures facilitated an understanding of their effect (or lack thereof). This was found to be conducive to generating public support for infection control measures.
5	Providing data “close to home”	To inform individuals of risks in their immediate surroundings, granular geographic breakdowns are needed. Data that are highly aggregated are difficult to understand. Maps (over tables and charts) were most effective to provide geographic information.
6	Disaggregating the information into relevant subgroups	Providing data with the possibility to explore varied population characteristics made indicators relatable to individual users. It enables understanding of risks and trends based on one’s own demographics. It can also facilitate equity-driven decision making by exposing differences among the population.
7	Using storytelling and visual cues	A concise narrative explaining the significance of a trend supports users to understand the importance of the information. Bare statistics without a narrated analysis leave the burden of interpretation solely to the user. Brief explanations on the meaning of trends used in combination with visual techniques, such as intuitive color schemes and icons, supported ease of interpretation.

^a^As identified by [28].

Two individual researchers (VB and TJ) marked these as either not present, somewhat present, or clearly present and provided argumentation for the ratings in free text. The actionability scoring was performed on December 17, 2020 (see Multimedia Appendix 2 for links to the archive). After their individual scorings, they shared and discussed their findings. Discrepancies in scorings were discussed between the 2 researchers and resulted in either an agreement for the score or an agreement to clarify discrepancies in the concluding qualitative appraisal for each of the 7 features.

### Appraisal Validation and Expert Reflections on the Decisions Made Throughout the Development Process of the COVID-19 Dashboard

A reflection meeting with the project team of the Ministry of Health, Welfare and Sport responsible for the COVID-19 dashboard was organized on April 23, 2021, with the following aims: to verify the appraisal of the dashboard’s actionability, to reflect on the development of the COVID-19 dashboard, and to understand decisions that were made towards the development of the dashboard. The project lead and 2 members of the team were present at the meeting. First, the study team presented the previously identified 7 key features of actionable dashboards. Second, the dashboard team shared their general reflection on the development of the dashboard from launch to date. Finally, the actionability appraisal of the 7 features of actionable dashboards were discussed in-depth. Due to COVID-19 restrictions, the meeting was conducted digitally. The meeting was moderated by NK, and notes were taken by TJ and VB. The Dutch dashboard scoring and appraisal for the 7 features were shared with the team in advance.

## Results

### Development of the COVID-19 Dashboard Over Time

Table 2 shows the findings of the descriptive assessment of the Dutch COVID-19 dashboard at 3 points in time.

**Table 2:** Descriptive checklist for the Dutch COVID-19 dashboard at 3 points in time.

Checklist items			Assessment date				
			1 (July 16, 2020)		2 (December 23, 2020)		3 (January 31, 2021)
**Purpose and users**							
	Purpose of dashboard use specified	Yes		Yes		Yes	
	Intended audience or users specified	No		No		No	
Content			2 themes, 10 indicators (80% epidemiological)		6 themes, 14 subthemes, and 59 indicators (78% epidemiological)		1 title page, 6 themes, 14 subthemes, and 63 indicators (79% epidemiological)
Indicator themes: subthemes (in order of occurrence on dashboard)			Intensive care admissions^a^, hospital admissions^a^, positive tested cases per 100,000 inhabitants^a^, reproduction number R^a^, amount of infectious people (estimated total and per 100,000 inhabitants)^a^; other data: number of patients for which GP^b^ suspects COVID-19 and sewage measurements of COVID-19 presence; nursing homes: amount of infections among nursing home residents, amount of nursing homes with at least one infection, deaths among nursing home residents		General: latest developments and active measures; infections: confirmed cases, infectious people, reproduction number, deaths; hospitals: hospital admissions and intensive care admissions; vulnerable groups: nursing care, disability care, people over 70 years old living at home; early signals: sewage water testing, GP-reported symptoms; behavior: compliance and behavior		Measures^a^; vaccinations; infections: confirmed cases, infectious people, reproduction number, deaths; hospitals: hospital admissions and intensive care admissions; vulnerable groups: nursing care, disability care, people over 70 years old living at home; early signals: sewage water testing, GP-reported symptoms; behavior: compliance and behavior
Data sources			Data sources specified: yes; data open-source: yes, most data; metadata specified: yes		Data sources specified: yes; data open-source: yes, most data; metadata specified: yes		Data sources specified: yes; data open-source: yes, most data; metadata specified: yes
**Analysis and display**							
	Time trends	Time trend analysis available: yes, after a few clicks; customizable: No		Time trend analysis available: yes, after a few clicks; customizable: yes		Time trend analysis available: yes, directly available; customizable: yes	
	Geographic levels of analysis	National: yes, full; safety regions: yes, very limited; municipalities: no		National: yes, full; safety regions: yes, partial; municipalities: yes, partial		National: yes, full; safety regions: yes, partial; municipalities: yes, partial	
	Disaggregation options	1 disaggregation option: long-term care facilities		4 disaggregation options: age, long-term care facilities, disability care facilities, over-70s living at home		4 disaggregation options: age, long-term care facilities, disability care facilities, over-70s living at home	
	Type of visualization	Maps, graphs, and charts to visualize indicators		Maps, graphs, and charts to visualize indicators		Maps, graphs, and charts to visualize indicators	
	Interpretation	Clarifying the quality of data: yes, in separate document and occasionally on dashboard itself; clarifying the meaning: yes, for some indicators; providing contextualization: no		Clarifying the quality of data: yes, in separate document and occasionally on dashboard itself; clarifying the meaning: yes, for almost every indicator; providing contextualization: no		Clarifying the quality of data: yes, in separate document and occasionally on dashboard itself; clarifying the meaning: yes, for every indicator; providing contextualization: yes, via news-like “stories”	
	Simplification techniques	Color coding: yes; icons: large icons for themes (eg, a head-and-test swab icon denoting testing indicators)		Color coding: yes; icons: large icons for themes (eg, a head-and-test swab icon denoting testing indicators) and small icons (eg, up and down arrows for changes)		Color coding: yes; icons: large icons for themes (eg, a head-and-test swab icon denoting testing indicators) and small icons (eg, up and down arrows for changes)	
	Interactive options	More information: yes; change of information: no; change of display: no		More information: yes; change of information: yes; change of display: yes		More information: yes; change of information: yes; change of display: yes	

^a^Presented as subthemes.

^b^GP: general practitioner.

#### Purpose and Users

A general statement of purpose was found at all 3 points in time. The statement highlighted the severity of the COVID-19 pandemic and explained the need to create a dashboard in order to use information to monitor the virus and to ensure that measures would be taken only where appropriate. The statement also emphasized that a political assessment of all relevant aspects was to include broader social and economic interests (see Multimedia Appendix 1 for archives). The target audience for the dashboard was not explicitly stated.

#### Content and Data

The number of indicators available on the dashboard increased over time, from 10 to 63 indicators. Both new themes were included (such as behavior and vaccinations), and the level of detail and stratification of previously available indicator themes was expanded (such as deaths, with an added indicator of excess mortality). The dashboard’s focus was mainly on epidemiological indicators (at different time points: 80%, 78%, and 79%, respectively), such as infection incidence numbers, reproduction rate, estimated number of infectious persons, and deaths. Health system capacity indicators included COVID-19 patients (such as numbers admitted to hospitals) and did not address non-COVID care needs. During our study, 4 social and behavioral indicators were added (added from December 10, 2020), such as percentage supporting wearing masks in public transport and percentage adhering to wearing a mask in public transport. These indicators showed self-reported survey outcomes on the compliance with and support for the COVID-19 measures taken by the government, based on data collected by the RIVM. No indicators relating to the social and economic impacts of the pandemic were observed.

Data sources were noted at all 3 points in time, and the data used in the dashboard were largely open-source. Metadata were provided in a separate document.

#### Analysis and Display of Data

The time trend information became more detailed over time. Customizable time trends and comparisons of indicator outcomes with outcomes for previous weeks, days, 2-day periods, or 3-day periods were provided. Geographic information became more granular over time. For instance, additional indicators on municipal and safety region levels alongside the primarily available national level were provided at the last 2 assessment dates. Disaggregation options were also expanded over time, as age groups and multiple vulnerable groups (eg, disability care homes and people over 70 years old living at home) were added.

Although visualization options were not adapted, during the second and third descriptive assessments, we observed additional interactive options providing change of information and display and more use of simplification techniques through small icons. For example, absolute numbers of infections and a relative number per 100,000 inhabitants were used at all 3 time points of the descriptive assessment. At all 3 time points, a scale was used to visualize the relative numbers, but at the last 2 time points, interpretation of these numbers was simplified by adding a norm value and color coding (red colors for “bad” and green colors for “good”) to the scale.

### Actionability of the COVID-19 Dashboard

Table 3 provides the initial researcher ratings and our consolidated appraisal of the Dutch government’s COVID-19 dashboard, version December 17, 2020, in terms of the 7 established features of highly actionable COVID-19 dashboards.

**Table 3:** Common features of highly actionable COVID-19 dashboards: appraisal of the Dutch government dashboard, version December 17, 2020.

Feature	Assessment				Combined qualitative assessment of the researchers
	Not present	Somewhat present	Clearly present		
Knowing the audience and its information needs	0	2	0	The dashboard’s purpose is stated, and, because it is a public website, the general public is implicitly the audience. That is not explicitly stated, however, nor is the dashboard sufficiently adapted to the audience, as professional jargon limits usability by the general public.	
Managing the type, volume, and flow of information	0	1	1	The volume of information necessitates clicking and scrolling multiple times to view dashboard components. However, 2 types of navigation options simplify information searches: (1) 3 tabs for national, safety region, and municipal geographic disaggregation; (2) a left-hand menu to click on indicator themes or subthemes.	
Making data sources and methods clear	0	0	2	Data sources and methods are explicitly noted per indicator, and many datasets are open-access.	
Linking time trends to policy and policy decisions	2	0	0	Time trends are displayed, but there are no links from time trends to policy decisions. Solely the current policy measures are specified, together with the current risk levels by safety region.	
Providing data “close to home”	0	2	0	The dashboard provides information down to municipal levels. Neighborhood-level information or information by postal code is not given.	
Breaking down the population into relevant subgroups	0	2	0	Breakdowns of information are available for (1) age (only for current daily new infections) and (2) 3 vulnerable groups: nursing home residents, disability facility residents, and people over 70 years old living at home. Disaggregation of information by gender, socioeconomic status, or ethnicity is not available.	
Using storytelling and visual cues	0	1	1	Narratives are present and explain why an indicator is presented and how it should be interpreted. However, interlinkages between indicators are not available. Visual cues such as color schemes and icons are used to show an indicator’s outcome severity. Information volumes and wordings are not adapted for easy navigation by the general public.	

### Decisions Made in the Development of the COVID-19 Dashboard by its Developers

Various reflections were made in the meeting with the COVID-19 dashboard team of the Ministry of Health, Welfare and Sport in the spring of 2021. The following sections include the considerations the dashboard development team made in their decisions to shape the COVID-19 dashboard, ordered by the 7 features of actionable dashboards.

#### Knowing the Audience and Their Information Needs

At the outset of the dashboard, policy makers, notably in the 25 safety regions, were the primary target audience. Even though much of the same indicators were used over time, there is currently a different medium to provide daily updates to policy makers. Thus, the dashboard was found to be gradually adjusted to addressing the general public. The dashboard has been designed for the purpose of high-frequency (daily) updates in a focused area, which, from the perspective of the COVID-19 dashboard team, is less fitting for the purpose of more broadly reporting on (health) systemwide performance to a wider public. The data infrastructure of the dashboard can be re-used in future situations in which high-frequency daily data updates might be necessary for reporting on other (health) system performance areas.

#### Managing the Type, Volume, and Flow of the Information

The letter to the House of Representatives in May 2020 has been the leading document in guiding choices on type, volume, and flow of information displayed on the COVID-19 dashboard. The information on the dashboard was found to be organized in chronological order of the infection and successive COVID-19 disease process (estimate infected persons, COVID-19 positive test results, hospital admissions, intensive care unit admission, deaths). To increase quick access to the most important information, a homepage was added when the audience of the dashboard changed from policy makers to the broader public. To facilitate navigation, visits to the different subtabs of the dashboards were reported to be monitored. When a certain subtheme had become more popular, the sidebar could be amended to prioritize that particular subject. For example, when the vaccination subtab was visited more often, it was moved to the top of the page.

#### Making Data Sources and Methods Clear

It was a political decision to foster transparency in data sources and methods. To ensure that the data sources and methods would not yield discussion, transparency of data was maximized by making it largely available as open source.

#### Linking Time Trends to Policy Decisions

The dashboard development team was very careful not to link time trends to policy decisions. A caveat of linking policy decisions to time trends is that correlation is suggested. From the perspective of the dashboard development team, it was a deliberate choice not to make these assumptions. Instead, formal interpretations were handled by political decision makers, who were advised by multidisciplinary experts via the Outbreak Management Team.

#### Providing Data “Close to Home”

The dashboard team explained that, in some phases of the pandemic, as in the summer 2020 period when case numbers were low, more disaggregated information should ideally be provided in order to act locally to prevent the spread of the virus. However, the team did not have access to neighborhood-level data from all municipalities. Another consideration was that the more an individual zooms in at the local level, the lower the numbers become. For the perception of severity of the transmission and due to privacy issues, disaggregation could challenge its usefulness. The dashboard team did not think it was wise in this stage of the pandemic to invest in more locally available data. The team anticipated that in the future, when most of the population of the Netherlands is vaccinated, attention will turn to investing in information on the international status of the virus. Doing so may provide insights into possible mutation effects that can arise from international travel.

#### Breaking Down Population Data Into Relevant Subgroups

Disaggregating the data, for example by sex, has not been high on the agenda for the dashboard’s development. The dashboard team explained that data for socioeconomic status or ethnicity are not available in their database and would require integration of data on a personal identifier level, which was not available to the dashboard team.

#### Using Storytelling and Visual Cues

News-like items were added when the audience of the dashboard changed from policy makers to the broader public. This decision was made to enhance public understanding of the information provided, especially for visitors to the dashboard with less affinity towards numbers. During the development of the dashboard, ongoing research on public interpretation and understanding of different features of the dashboard were conducted in order to enhance actionability.

## Discussion

### Principal Findings

We assessed the COVID-19 dashboard provided by the Dutch government in terms of its development during the pandemic and its actionability for conveying information on the pandemic and supporting data-driven decision making. We found that the dashboard predominantly showed epidemiological information on COVID-19. It had been developed and adapted by adding more in-depth indicators, more geographic disaggregation options, and new indicator themes. It also changed in target audience from policy makers to the general public; thus, a homepage was added with the most important information, using news-like items to explain the provided indicators and conducting research to enhance public understanding of the dashboard.

Two of the 4 key components advised by the WHO Regional Office for Europe for monitoring and managing the pandemic were still missing from the dashboard 10 months after the first patient with COVID-19 was reported in the Netherlands: (1) indicators of available capacity for dual-track health system management to safeguard non-COVID care and (2) indicators of social and economic impact. As COVID-19 care needs draw on limited health system resources, non-COVID care needs must also be taken into account when weighing options to take policy decisions [32,33]. Although information on health care service utilization for other conditions (dual-track monitoring) as well as socioeconomic impact is available via a variety of other government-linked organizations—including the Dutch Health Authority (NZA), Statistics Netherlands (CBS), and the Netherlands Institute for Social Research (SCP)—such information is not provided on the COVID-19 dashboard, thus constraining an overall assessment of the situation. As the pandemic persists, the monitoring of both non-COVID care access and socioeconomic impact is of growing importance to adequately inform policy decisions [11]. Several Dutch political parties have emphasized the need to obtain and discuss such information in a more “overall” format.

Experiences from other countries have shown the importance of relevant disaggregation into subgroups, such as socioeconomic status and ethnicity, in order to highlight inequalities within populations [34-38]. Reported data from the United States on negative changes in life expectancy and the substantial differences between ethnic groups have underscored the relevance of transparent, differential data [39,40]. Ethnicity is not represented in the established data infrastructure on health; thus, the Dutch COVID-19 dashboard does not provide this disaggregation option. In the Netherlands, cohort studies are used to gain insights into ethnic inequalities [41], but such studies carry significant time delays for accessing valuable information needed at the time of a pandemic. Two policy briefs released in May 2021 on the COVID-19 impact among ethnic groups underscore that disaggregating data on COVID-19 impact for ethnic groups can lead to valuable insights for policy decision making [42,43]. The dashboard development team also noted that disaggregation of data by sex and gender was not high on their agenda. COVID-19 outcomes have shown to be affected by sex and gender differences, for example in increased mortality in men compared with women [44]. Taking into account data on sex and gender is needed in order to distinguish potential sex-based immunological differences or gender-based differences in behavior that require tailored care or policy actions [45,46].

The dashboard requires timely data to inform the public of the constantly evolving context, as was the case with the indicators on administered vaccinations and percentages of infected individuals with mutated virus variants. Communicating a constantly changing message to a wide public is complex. Dashboards therefore need to promptly adapt and evolve so as to take the most important considerations of “today” into account, while also providing “stable” trend indicators to highlight the changing circumstances. When data reporting or data infrastructure lagged behind, the Dutch government chose in many instances to show estimated or not yet contextualized data, for example on estimated numbers of infectious people or on sewage water testing [47]. By our third assessment of the dashboard, news-like text items had been added to bridge gaps in still-unavailable data and to increase public understanding of the provided indicators, making use of textual explanations such as what impact the new variant might have and why caution was needed. Experts are divided as to whether it is wise to publish work-in-progress data for indicators such as sewage water testing. Some ask why an indicator should be shown if it does not lead to consequences in policy decisions [48], while others argue that it could provide early warnings to the general public [49,50]. Experts in health communication emphasize that information should be presented in a way that assumes that citizens have a choice in their behavior [51,52]. Then, a dashboard can not only communicate a general picture of fear but also acknowledge the freedom of individuals to make a choice, which appeals to their responsibility for the collective.

The duration of the COVID-19 pandemic and the pressing need for immediate action have exposed weaknesses in the health and social care data infrastructure of the Netherlands in terms of its timeliness and accessibility to (and interoperability of) health and social care data and socioeconomic data for the benefit of public health. These gaps are evident in the limited availability of timely dual-track indicators, data on social and economic impact, and possibilities for relevant population breakdowns. We advise (1) establishing timely indicators relating to health system capacity in the Dutch health information infrastructure, (2) including relevant data disaggregation options, and (3) enabling interoperability between social, health, and economic data sources.

### Strengths and Limitations

A particular strength of this study is that the checklist and the features of actionable dashboards that we applied were developed on the basis of a large sample of international COVID-19 dashboards (158 dashboards in 53 countries) [28]. A number of limitations has to be taken into account in interpreting the presented findings. Both the pandemic and the dashboard are constantly developing. As 3 discrete points in time were chosen for the descriptive checklist, some back-and-forth changes to the dashboard between time points could have been missed. Another limitation is that, although the same researcher (VB) filled out the checklist at all 3 time points, the archiving of the dashboard during the first checklist was limited to a copy of its main page, so that the in-depth tabs could not be revisited and checked by other researchers.

### Conclusion

The Dutch COVID-19 dashboard developed over time to be fit for purpose and fit for use in terms of providing epidemiological information to the general public as the target audience. This can help to transform the dashboard from a tool of public accountability to an instrument for community action to contain the pandemic. However, to enhance its actionability for monitoring COVID-19 and its social and economic impact, including a broader set of indicators may be considered. Therefore, gaps in the Dutch health information infrastructure need to be addressed. To strengthen the Dutch health system’s ability to cope with upcoming phases of the COVID-19 pandemic or future public health emergencies, we advise (1) establishing timely indicators related to health system capacity, (2) including relevant data disaggregation options (eg, sex, socioeconomic status), and (3) enabling interoperability between social, health, and economic data sources.

## Appendix

Multimedia Appendix 1 Archived copies of the Dutch COVID-19 government dashboard.

Multimedia Appendix 2 Descriptive checklist.

## Abbreviations

CBS: Statistics Netherlands
NZA: Dutch Healthcare Authority
RIVM: National Institute for Public Health and the Environment
SARS-CoV-2: severe acute respiratory syndrome coronavirus 2
SCP: Netherlands Institute for Social Research
WHO: World Health Organization

## References

[1] Jakab M, Nathan NL, Pastorino G, Evetovits T, Garner S, Langins M, Scotter C, Azzopardi-Muscat N (2020). Managing health systems on a seesaw: balancing the delivery of essential health services whilst responding to COVID-19. Eurohealth.

[2] Dong E, Du H, Gardner L (2020). An interactive web-based dashboard to track COVID-19 in real time. The Lancet Infectious Diseases.

[3] Wissel B, Van Camp PJ, Kouril M, Weis C, Glauser T, White P, Kohane IS, Dexheimer JW (2020). An interactive online dashboard for tracking COVID-19 in U.S. counties, cities, and states in real time. J Am Med Inform Assoc.

[4] Berry I, Soucy JR, Tuite A, Fisman D, COVID-19 Canada Open Data Working Group (2020). Open access epidemiologic data and an interactive dashboard to monitor the COVID-19 outbreak in Canada. CMAJ.

[5] West R, Michie S, Rubin GJ, Amlôt R (2020). Applying principles of behaviour change to reduce SARS-CoV-2 transmission. Nat Hum Behav.

[6] Tomes N (2020). Managing the modern infodemic. CMAJ.

[7] Bavel JJV, Baicker K, Boggio PS, Capraro V, Cichocka A, Cikara M, Crockett MJ, Crum AJ, Douglas KM, Druckman JN, Drury J, Dube O, Ellemers N, Finkel EJ, Fowler JH, Gelfand M, Han S, Haslam SA, Jetten J, Kitayama S, Mobbs D, Napper LE, Packer DJ, Pennycook G, Peters E, Petty RE, Rand DG, Reicher SD, Schnall S, Shariff A, Skitka LJ, Smith SS, Sunstein CR, Tabri N, Tucker JA, Linden SVD, Lange PV, Weeden KA, Wohl MJA, Zaki J, Zion SR, Willer R (2020). Using social and behavioural science to support COVID-19 pandemic response. Nat Hum Behav.

[8] Shelton T (2020). A post-truth pandemic?. Big Data & Society.

[9] Everts J (2020). The dashboard pandemic. Dialogues in Human Geography.

[10] Rocha R (2020). The flurry of daily pandemic data can be overwhelming: Here's how to make sense of it. CBC News.

[11] (2020). Strengthening and adjusting public health measures throughout the COVID-19 transition phases: Policy considerations for the WHO European Region. World Health Organization.

[12] HealthPros - International Training Network for Healthcare Performance Intelligence Professionals.

[13] Ivanković D, Barbazza E, Bos V, Brito F, Jamieson Gilmore K, Jansen T, Kara P, Larrain N, Lu S, Meza-Torres B, Mulyanto J, Poldrugovac M, Rotar A, Wang S, Willmington C, Yang Y, Yelgezekova Z, Allin S, Klazinga N, Kringos D (2021). Features Constituting Actionable COVID-19 Dashboards: Descriptive Assessment and Expert Appraisal of 158 Public Web-Based COVID-19 Dashboards. J Med Internet Res.

[14] Barbazza E, Klazinga NS, Kringos DS (2021). Exploring the actionability of healthcare performance indicators for quality of care: a qualitative analysis of the literature, expert opinion and user experience. BMJ Qual Saf.

[15] Barbazza E, Ivanković D, Wang S, Gilmore KJ, Poldrugovac M, Willmington C, Larrain N, Bos V, Allin S, Klazinga N, Kringos D (2021). Exploring changes to the actionability of COVID-19 dashboards over the course of 2020 in the Canadian context: descriptive assessment and expert appraisal study. J Med Internet Res.

[16] (2020). Expert tafel Lessons Learned - Thema Dashboard. Ministry of Health, Welfare and Sport.

[17] (2020). Rapportage kleuren corona escalatieladder. Rijksoverheid.

[18] (2020). Corona Dashboard linkermenu. Ministry of Health, Welfare and Sport.

[19] Dutch COVID-19 dashboard. Ministry of Health, Welfare and Sport.

[20] Kamerbrief eerste COVID-19 patiënt in Nederland. Rijksoverheid.

[21] Current situation in the Netherlands. Rijksoverheid.

[22] Data on country response measures to COVID-19. European Centre for Disease Prevention and Control.

[23] Coronavirus COVID-19. Rijksoverheid.

[24] Brief over advies van Outbreak Management Team over COVID-19. Rijksoverheid.

[25] Actuele informatie over COVID-19. Rijksinstituut voor Volksgezondheid en Milieu.

[26] Statistics Netherlands (CBS) Well-being in times of corona.

[27] Kamerbrief COVID 19: Update stand van zaken. Rijksoverheid.

[28] Ivanković D, Barbazza E, Bos V, Brito Fernandes Ó, Jamieson Gilmore K, Jansen T, Kara P, Larrain N, Lu S, Meza-Torres B, Mulyanto J, Poldrugovac M, Rotar A, Wang S, Willmington C, Yang Y, Yelgezekova Z, Allin S, Klazinga N, Kringos D (2021). Features Constituting Actionable COVID-19 Dashboards: Descriptive Assessment and Expert Appraisal of 158 Public Web-Based COVID-19 Dashboards. J Med Internet Res.

[29] Lasswell H, Bryson L (1948). The structure and function of communication in society. The Communication of Ideas.

[30] Kringos D, Carinci F, Barbazza E, Bos V, Gilmore K, Groene O, Gulácsi L, Ivankovic D, Jansen T, Johnsen SP, de Lusignan S, Mainz J, Nuti S, Klazinga N, HealthPros Network (2020). Managing COVID-19 within and across health systems: why we need performance intelligence to coordinate a global response. Health Res Policy Syst.

[31] Kringos DS, Groene O, Johnsen SP (2019). Training the first generation of health care performance intelligence professionals in Europe and Canada. Acad Med.

[32] Erondu NA, Martin J, Marten R, Ooms G, Yates R, Heymann DL (2018). Building the case for embedding global health security into universal health coverage: a proposal for a unified health system that includes public health. Lancet.

[33] Kluge HHP, Wickramasinghe K, Rippin HL, Mendes R, Peters DH, Kontsevaya A, Breda J (2020). Prevention and control of non-communicable diseases in the COVID-19 response. The Lancet.

[34] van Dorn A, Cooney RE, Sabin ML (2020). COVID-19 exacerbating inequalities in the US. Lancet.

[35] Chen J, Krieger N (2021). Revealing the unequal burden of COVID-19 by income, race/ethnicity, and household crowding: US county versus zip code analyses. J Public Health Manag Pract.

[36] Patel J, Nielsen F, Badiani A, Assi S, Unadkat V, Patel B, Ravindrane R, Wardle H (2020). Poverty, inequality and COVID-19: the forgotten vulnerable. Public Health.

[37] Bibby J, Everest G, Abbs I (2020). Will COVID-19 be a watershed moment for health inequalities?. The Health Foundation.

[38] Marmot M (2020). Society and the slow burn of inequality. The Lancet.

[39] Webb Hooper M, Nápoles AM, Pérez-Stable EJ (2020). COVID-19 and Racial/Ethnic Disparities. JAMA.

[40] Andrasfay T, Goldman N (2021). Reductions in 2020 US life expectancy due to COVID-19 and the disproportionate impact on the Black and Latino populations. Proc Natl Acad Sci U S A.

[41] Stronks K, Snijder MB, Peters RJ, Prins M, Schene AH, Zwinderman AH (2013). Unravelling the impact of ethnicity on health in Europe: the HELIUS study. BMC Public Health.

[42] Stronks K, Prins M, Agyemang C (2021). Bevolkingsgroepen met Migratieachtergrond Zwaarder Getroffen Door COVID-19. Coronatijden in Nederland.

[43] Torensma M, Skowronek N, De LT, Van DMM, Stronks K De positie van ongedocumenteerde arbeidsmigranten in de COVID-19 crisis: lessen uit onderzoek voor beleid en praktijk. Coronatijden in Nederland.

[44] Pérez-López FR, Tajada M, Savirón-Cornudella R, Sánchez-Prieto M, Chedraui P, Terán E (2020). Coronavirus disease 2019 and gender-related mortality in European countries: A meta-analysis. Maturitas.

[45] Wenham C, Smith J, Morgan R (2020). COVID-19: the gendered impacts of the outbreak. The Lancet.

[46] Brady E, Nielsen MW, Andersen JP, Oertelt-Prigione S (2021). Lack of consideration of sex and gender in COVID-19 clinical studies. Nat Commun.

[47] Leenen I Expert-reflectie ten behoeve van Lessons Learned COVID-19. Rijksoverheid.

[48] Blauw S Maak een dashboard voor alle Nederlanders. Rijksoverheid.

[49] Slagter B Expert-reflectie ten behoeve van Lessons Learned COVID-19. Rijksoverheid.

[50] van Zelst M Expert-reflectie ten behoeve van Lessons Learned COVID-19. Rijksoverheid.

[51] de Vries M, Claassen L, te Wierik MJM, van den Hof S, Brabers AE, de Jong JD, Timmermans DR, Timen A (2021). Dynamic Public Perceptions of the Coronavirus Disease Crisis, the Netherlands, 2020. Emerg Infect Dis.

[52] Soetenhorst B (2021). Hoogleraar: prikpauzes AstraZeneca hadden voorkomen kunnen worden. Het Parool.

