# Supplementary material for: Development and Actionability of the Dutch COVID-19 Dashboard: Descriptive Assessment and Expert Appraisal Study
Source: JMIR Public Health Surveill. 2021 Oct 12;7(10):e31161. doi: 10.2196/31161 (PMC8513744; doi:10.2196/31161)
Supplement: Multimedia Appendix 1 [file publichealth_v7i10e31161_app1.docx]

Development of the Dutch COVID-19 Dashboard: Descriptive Assessment and Expert Appraisal of its Fitness for Purpose and Use

**Multimedia Appendix 1:** Archived copies of the Dutch COVID-19 government dashboard

Archive for July 16, 2020 assessment: https://archive.vn/lBh0Q

| **Title Webpage** | **URL** | **Archive Dec 17, 2020** | **Archive Dec 23, 2020** | **Archive Jan 31, 2021** |
| --- | --- | --- | --- | --- |
| Homepage | https://coronadashboard.rijksoverheid.nl | https://archive.vn/I02rX | https://archive.vn/0rEaI | https://archive.vn/TY7Vi |
| Measures | https://coronadashboard.rijksoverheid.nl/landelijk/maatregelen | Not applicable | Not applicable | https://archive.vn/sHGmx |
| About this dashboard | https://coronadashboard.rijksoverheid.nl/over | https://archive.vn/FKWTV | https://archive.vn/HvuHv | https://archive.vn/3vyPa |
| Indicator accountability | https://coronadashboard.rijksoverheid.nl/verantwoording | https://archive.vn/NNvU7 | Available upon request at research group | Available upon request at research group |
| Infections – positive tests | https://coronadashboard.rijksoverheid.nl/landelijk/positief-geteste-mensen | https://archive.vn/sT8Wt | https://archive.vn/OcFDE | https://archive.vn/HBFAB |
| Infections – infectious persons | https://coronadashboard.rijksoverheid.nl/landelijk/besmettelijke-mensen | https://archive.vn/jXoId | https://archive.vn/WfeEQ | https://archive.vn/LMjDn |
| Infections – R | https://coronadashboard.rijksoverheid.nl/landelijk/reproductiegetal | https://archive.vn/qXiII | https://archive.vn/TfJqo | https://archive.vn/1IXR8 |
| Infections – mortality | https://coronadashboard.rijksoverheid.nl/landelijk/sterfte | https://archive.vn/ndRxV | https://archive.vn/msfJO | https://archive.vn/3OhTw |
| Hospitals – hospital admissions | https://coronadashboard.rijksoverheid.nl/landelijk/ziekenhuis-opnames | https://archive.vn/pKori | https://archive.vn/vxtLH | https://archive.vn/VUcFX |
| Hospitals - ICU admissions | https://coronadashboard.rijksoverheid.nl/landelijk/intensive-care-opnames | https://archive.vn/rwZok | https://archive.vn/OjnYb | https://archive.vn/g39Yb |
| Vulnerable groups – nursing homes | https://coronadashboard.rijksoverheid.nl/landelijk/verpleeghuiszorg | https://archive.vn/b1x1y | https://archive.vn/IuCL0 | https://archive.vn/9sNOY |
| Vulnerable groups – disability | https://coronadashboard.rijksoverheid.nl/landelijk/gehandicaptenzorg | https://archive.vn/Et8oD | https://archive.vn/ATgCN | https://archive.vn/ZL7En |
| Vulnerable groups – over 70 living at home | https://coronadashboard.rijksoverheid.nl/landelijk/thuiswonende-ouderen | https://archive.vn/gDoWj | https://archive.vn/thUtA | https://archive.vn/gLqTP |
| Early signals – Sewage water measures | https://coronadashboard.rijksoverheid.nl/landelijk/rioolwater | https://archive.vn/w1f23 | https://archive.vn/8RS7H | https://archive.vn/p9nZ4 |
| Early signals – symptoms at GP | https://coronadashboard.rijksoverheid.nl/landelijk/verdenkingen-huisartsen | https://archive.vn/uY7p0 | https://archive.vn/NpcK7 | https://archive.vn/1yIBp |
| Behaviour – compliance and behaviour | https://coronadashboard.rijksoverheid.nl/landelijk/gedrag | https://archive.vn/n7Hc8 | https://archive.vn/RzQNT | https://archive.vn/hvmQa |
| Safety regions | https://coronadashboard.rijksoverheid.nl/veiligheidsregio | https://archive.vn/nlZNH | https://archive.vn/fy33Q | https://archive.vn/evRBI |
| Safety region: Amsterdam-Amstelland | https://coronadashboard.rijksoverheid.nl/veiligheidsregio/VR13/positief-geteste-mensen | https://archive.vn/38SJe | https://archive.vn/4PISy | https://archive.vn/Gev2s |
| Municipalities | https://coronadashboard.rijksoverheid.nl/gemeente | https://archive.vn/gl5Nc | https://archive.vn/T6nHg | https://archive.vn/iGvEu |
| Municipalities: Amsterdam | https://coronadashboard.rijksoverheid.nl/gemeente/GM0363/positief-geteste-mensen | https://archive.vn/Hay0S | https://archive.vn/80mVk | https://archive.vn/8ZPtT |
| Vaccinations | https://coronadashboard.rijksoverheid.nl/landelijk/vaccinaties | Not applicable | Not applicable | https://archive.vn/7xI4j |
